# Prediagnostic Immune Cell Profiles and Breast Cancer

**DOI:** 10.1001/jamanetworkopen.2019.19536

**Published:** 2020-01-17

**Authors:** Jacob K. Kresovich, Katie M. O’Brien, Zongli Xu, Clarice R. Weinberg, Dale P. Sandler, Jack A. Taylor

**Affiliations:** 1 Epidemiology Branch, National Institute of Environmental Health Sciences, National Institutes of Health, Research Triangle Park, North Carolina; 2 Biostatistics and Computational Biology Branch, National Institute of Environmental Health Sciences, National Institutes of Health, Research Triangle Park, North Carolina; 3 Epigenetic and Stem Cell Biology Laboratory, National Institute of Environmental Health Sciences, National Institutes of Health, Research Triangle Park, North Carolina

## Abstract

**Importance:**

Higher overall leukocyte counts in women may be associated with increased risk of breast cancer, but the association of specific leukocyte subtypes with breast cancer risk remains unknown.

**Objective:**

To determine associations between circulating leukocyte subtypes and risk of breast cancer.

**Design, Setting, and Participants:**

Between 2003 and 2009, the Sister Study enrolled 50 884 women who had a sister previously diagnosed with breast cancer but were themselves breast cancer free. A case-cohort subsample was selected in July 2014 from the full Sister Study cohort. Blood samples were obtained at baseline, and women were followed up through October 2016. Data analysis was performed in April 2019.

**Main Outcomes and Measures:**

The main outcome was the development of breast cancer in women. Whole-blood DNA methylation was measured, and methylation values were deconvoluted using the Houseman method to estimate proportions of 6 leukocyte subtypes (B cells, natural killer cells, CD8^+^ and CD4^+^ T cells, monocytes, and granulocytes). Leukocyte subtype proportions were dichotomized at their population median value, and Cox proportional hazard models were used to estimate associations with breast cancer.

**Results:**

Among 2774 non-Hispanic white women included in the analysis (mean [SD] age at enrollment, 56.6 [8.8] years), 1295 women were randomly selected from the full cohort (of whom 91 developed breast cancer) along with an additional 1479 women who developed breast cancer during follow-up (mean [SD] time to diagnosis, 3.9 [2.2] years). Circulating proportions of B cells were positively associated with later breast cancer (hazard ratio [HR], 1.17; 95% CI, 1.01-1.36; *P* = .04). Among women who were premenopausal at blood collection, the association between B cells and breast cancer was significant (HR, 1.38; 95% CI, 1.05-1.82; *P* = .02), and an inverse association for circulating proportions of monocytes was found (HR, 0.75; 95% CI, 0.57-0.99; *P* = .05). Among all women, associations between leukocyte subtypes and breast cancer were time dependent: higher monocyte proportions were associated with decreased near-term risk (within 1 year of blood collection, HR, 0.62; 95% CI, 0.43-0.89; *P* = .01), whereas higher B cell proportions were associated with increased risk 4 or more years after blood collection (HR, 1.38; 95% CI, 1.15-1.67; *P* = .001).

**Conclusions and Relevance:**

Circulating leukocyte profiles may be altered before clinical diagnoses of breast cancer and may be time-dependent markers for breast cancer risk, particularly among premenopausal women.

## Key Points

**Question:**

Is there an association between specific subtypes of circulating leukocytes and the risk of breast cancer?

**Findings:**

In this case-cohort study of 2774 women, lower proportions of circulating monocytes were associated with a higher risk of breast cancer within 1 year of the blood collection, whereas higher proportions of circulating B cells were associated with a higher risk of breast cancer 4 or more years later.

**Meaning:**

Shifts in circulating leukocyte profiles appear to precede a breast cancer diagnosis and may serve as markers of time-dependent breast cancer risk.

## Teaser

This case-cohort study examines the associations between proportions of circulating leukocyte subtypes and the risk of breast cancer.

## Introduction

The immune system plays conflicting roles in cancer development and progression. Although immunosurveillance provides an important first defense against neoplastic cells, immune responses can also be associated with tumor growth by altering tissue microenvironments and selecting more-virulent cells through immunoediting.^1,2^ Local immune cell responses to breast tumors are well characterized and are associated with cancer progression and survival.^3,4,5,6,7^ Peripheral blood leukocyte profiles measured after diagnosis may also be associated with prognosis,^8,9,10^ but whether leukocyte subtypes are altered before diagnosis remains largely unexplored.

Prospective epidemiologic studies^11,12,13^ suggest that women with higher overall leukocyte counts, or certain autoimmune disorders, may be at increased risk of breast cancer. However, leukocytes are diverse and can be broadly defined by their cell lineage (myeloid and lymphoid); these subtypes may be differentially associated with breast cancer. For example, increasing proportions of myeloid-lineage subtypes have been reported to contribute to tumor development.^14,15,16^ Few large-scale studies have assessed immune cell profiles; isolation of leukocyte subtypes in peripheral blood has traditionally required flow cytometry, which is an expensive and time-consuming assay that requires fresh blood samples.^17^ Thus, studies of breast cancer and leukocyte subtypes have been limited to small case-control studies^18,19,20^ using case samples obtained at diagnosis.

Leukocyte subtypes can be distinguished by their lineage-specific patterns of DNA methylation, allowing estimation of circulating leukocyte composition using blood DNA.^21,22,23,24,25,26^ Epigenome-wide methylation array data are increasingly available in large epidemiologic studies and can be deconvoluted to estimate proportions of common leukocyte subtypes.^21,22^ Using such deconvolution, methylation-derived leukocyte profiles, including the methylation-derived neutrophil-to-lymphocyte ratio (mdNLR), have been shown to be altered in patients with a variety of cancers and appear to be associated with cancer prognosis.^27,28,29,30,31,32,33^ Recently, among a small population of heavy smokers, the mdNLR was reported to be altered years before a lung cancer diagnosis, supporting the hypothesis that circulating leukocyte profiles may be markers of cancer risk.^34^ Here, we use prospectively collected peripheral blood samples from a large, nationwide cohort of women and report associations between methylation-derived leukocyte profiles and breast cancer risk.

## Methods

### Study Population

The Sister Study^35^ enrolled a cohort of 50 884 women residing in the United States or Puerto Rico between 2003 and 2009. Eligible women were aged 35 to 75 years and could not have had breast cancer themselves but must have had a biological sister who received a diagnosis of breast cancer. Enrolled women are recontacted annually by email, mail, or telephone and are asked to complete either a short questionnaire about recent diagnoses, including any cancer diagnosis, or a more comprehensive questionnaire (every third year) on changes in health, lifestyle, and exposures. The annual response rate has consistently been greater than 90%. Women reporting a breast cancer diagnosis are contacted 6 months after diagnosis and asked to provide authorization to retrieve medical records.

A case-cohort subsample of women was selected for whole-blood DNA methylation analysis (eFigure 1 in the Supplement).^36^ To avoid possible confounding by ancestry, our analysis was restricted to white women who self-reported as non-Hispanic. For the subcohort, approximately 3% (1336) of eligible non-Hispanic white women were randomly selected from the full cohort (ie, the random subcohort). The random subcohort is meant to be representative of all non-Hispanic white women in the cohort. Of these 1336 women, 91 received a diagnosis of breast cancer after enrollment but before October 2016 (data release 6.0). As our case set, we selected the remaining eligible 1540 non-Hispanic white women who received a diagnosis of ductal carcinoma in situ (DCIS) or invasive breast cancer during the time between enrollment and when the case-cohort was sampled (July 2014). Person-time of the women selected into the case-cohort was weighted back to the population of non-Hispanic white women from the full Sister Study cohort.^37^ Data analysis was conducted in April 2019.

Written informed consent and blood samples were obtained during a home visit at enrollment. The study was approved by the institutional review boards of the National Institute of Environmental Health Sciences and the Copernicus Group. This study follows the Strengthening the Reporting of Observational Studies in Epidemiology (STROBE) reporting guideline.

### Temporal Stability of Leukocyte Subtypes

Although the stability of DNA methylation at individual CpG sites included on the HumanMethylation450 BeadChip has been examined,^38,39,40^ comparatively less is known about the stability of the methylation-inferred leukocyte proportions. Examining the variability of the subtype proportions over time could provide evidence for the robustness of their associations with breast cancer risk. To examine the stability of the subtypes over a 1-year period, serial samples of whole blood were collected from an independent set of 8 cancer-free women. These samples were handled and processed with the same procedures as the Sister Study. For each woman, samples from 3 time points, approximately 5 months apart (January or February, May, and October 2008), were selected for analysis (total of 24 samples).

### Genomic DNA Processing and Immune Cell Deconvolution

The DNA processing procedures have been described elsewhere.^41,42^ In brief, DNA was extracted from whole blood, and 1 μg was bisulfite-converted in 96-well plates using the EZ DNA Methylation Kit (Zymo Research). Samples were tested for completion of bisulfite conversion and, according to the manufacturer’s protocol, analyzed on Infinium HumanMethylation450 BeadChips (Illumina). For the independent sample used to examine the stability of the distribution of leukocyte subtypes over time, methylation was measured using an older microarray, the Infinium HumanMethylation27 BeadChip (Illumina). Methylation analysis was performed at the National Institutes of Health Center for Inherited Disease Research. The arrays were processed using high-throughput robotics to minimize batch effects. Methylation data preprocessing and quality control were completed using the ENmix method^43^ in R statistical software version 3.6.1 (R Project for Statistical Computing). This included reducing background noise with the ENmix method, correcting fluorescent dye bias using the RELIC method,^44^ quantile normalization to make overall array fluorescence intensity distribution comparable between arrays, and reducing Infinium I and II probe design bias using the regression on correlated probes method.^45^ We excluded 102 samples after quality control (61 cases and 41 noncases). Of these 102 samples, 91 had mean bisulfite intensity less than 4000 or had greater than 5% of probes with low-quality methylation values (detection *P* > .000001, <3 beads, or values outside of 3 times the interquartile range), 4 were outliers for their methylation beta value distributions, 1 had missing phenotype data, and 6 were from women whose date of diagnosis preceded blood collection.^46^ After excluding women whose blood methylation data failed quality control, there remained 1295 women from the random subcohort (of whom 91 developed breast cancer by October 2016) and 1479 women from the case set. Of the 1570 women with incident DCIS or invasive breast cancer who were included, pathology reports were obtained for 1539 (98%) of these women. Among women for whom we obtained pathology reports, the positive predictive value of a self-reported breast cancer was 99.4%.^47^

We used the Houseman method to statistically deconvolute distributions of 6 leukocyte subtypes: B cells, natural killer cells, and CD8^+^ and CD4^+^ T cells (lymphoid lineage), and monocytes and granulocytes (myeloid lineage).^21^ This deconvolution method has been validated using DNA methylation, and complete blood cell count data were assessed for the same blood collection.^22^ For each woman, the estimated proportions of the 6 cell types sum to 1. The mdNLR was calculated by dividing the estimated proportion of granulocytes by the sum of the lymphocyte proportions (CD8^+^ and CD4^+^ T cells, B cells, and natural killer cells).^32,34^ The leukocyte proportions and mdNLR estimates were then dichotomized at the median values for women in the random subcohort.

### Statistical Analysis

Comparing women who developed breast cancer with those who remained cancer free through follow-up, we described characteristics using means and SDs or counts and proportions. We tested for differences in baseline characteristics using 2-sample *t* tests for continuous and ordinal variables and χ^2^ tests for categorical variables. We further examined Spearman correlation coefficients (*r_s_*) between the leukocyte proportions. To test the stability of the distribution of leukocyte subtypes, intraclass correlation coefficients (ICCs) were calculated using 2-way mixed-effect models,^48^ treating the participant as a random effect and the visit at which the blood sample was collected as a fixed effect. To measure associations between the leukocyte subtypes and breast cancer risk, we used case-cohort Cox proportional hazard models with robust standard errors to calculate hazard ratios (HRs), 95% CIs, and 2-sided *P* values, with *P* ≤ .05 considered statistically significant.^37^ We excluded 1 DCIS case for whom age was missing at diagnosis. Because age was treated as the primary timescale for this analysis,^49^ all resulting HRs are, therefore, fully adjusted for age. In our primary analysis, we combined DCIS and invasive breast cancer diagnoses to represent all breast cancers. Menopause could affect leukocyte composition^50^; we therefore examined associations for women both overall and stratified by menopause status at blood collection and statistically tested for heterogeneity using an interaction term of the leukocyte subtype by menopause status. In follow-up analyses, we separately examined risks for DCIS and invasive breast cancer, treating the breast cancer subtype of interest as an event and otherwise censoring women at the time of their diagnosis or the end of follow-up (October 2016). We explored time dependence by examining whether leukocyte associations with breast cancer varied by the number of years between the blood collection and clinical diagnosis of breast cancer. Specifically, we estimated associations with breast cancer risk within 1 year of blood collection, 1 to less than 4 years after blood collection, and 4 years or more after blood collection.

We examined potential confounding by adjusting for established breast cancer risk factors measured at baseline, including menopause status (premenopause vs postmenopause), continuous versions of body mass index (calculated as the weight in kilograms divided by height in meters squared), physical activity, current alcohol intake, number of live births, age at first birth (among parous women), age at menarche, breastfeeding duration, and duration of hormone therapy and oral contraception use.^51,52,53,54,55^ The association between body mass index and breast cancer varies by menopause status^52^; we therefore included an interaction term between menopause and body mass index. Analyses were conducted using Stata statistical software version 15 (StataCorp).

## Results

### Sample Population

By design, all 2774 women sampled were non-Hispanic white (mean [SD] age at enrollment, 56.6 [8.8] years). The mean (SD) duration of follow-up was 6.0 (3.2) years. The women who developed breast cancer over follow-up tended to be older at blood collection (mean [SD] age, 57.7 [9.0] vs 55.1 [9.0] years), engage in less physical activity (mean [SD] number of metabolic equivalent tasks per week, 49.6 [30.0] vs 52.4 [32.0]), and be older at menopause onset (mean [SD] age, 50.7 [5.0] vs 49.6 [6.0] years) (Table 1). The mean (SD) time to diagnosis was 3.9 (2.2) years. Of the 1570 women who developed breast cancer, 1231 (78%) had invasive tumors.

**Table 1.** Participant Baseline Characteristics by Breast Cancer Status at the End of the Study Period in October 2016^a^

Characteristic	Cancer Status at Follow-up		*P* Value^b^
	Nonevent (n = 1204)	Event (n = 1570)	
Age, mean (SD), y	55.1 (9.0)	57.7 (9.0)	<.001
Alcohol, mean (SD), drinks/wk	2.9 (4.0)	3.3 (5.0)	.06
Physical activity, mean (SD), metabolic equivalent tasks/wk	52.4 (32.0)	49.6 (30.0)	.02
Hormone therapy use, mean (SD), y	3.8 (6.0)	4.8 (7.0)	<.001
Oral contraception use, mean (SD), y	6.0 (6.0)	5.9 (6.0)	.75
Parity, mean (SD), No. of total births	2.0 (1.0)	1.9 (1.0)	.65
Age, mean (SD), y			
At first birth^c^	24.7 (5.0)	25.0 (5.0)	.16
Menarche	12.6 (2.0)	12.6 (1.0)	.18
Menopause^d^	49.6 (6.0)	50.7 (5.0)	<.001
Education, No. (%)			
High school diploma or less	204 (17)	226 (14)	.06
Some college or college degree	717 (60)	924 (59)	
Advanced degree	283 (23)	420 (27)	
Body mass index, No. (%)^e^			
≤24.9 (underweight or normal)	482 (40)	591 (38)	.41
25-30 (overweight)	384 (32)	516 (33)	
>30 (obese)	336 (28)	463 (29)	
Missing	2	0	
Menopause status, No. (%)			
Premenopausal	408 (34)	418 (27)	<.001
Postmenopausal	795 (66)	1152 (73)	
Missing	1	0	
Stage at diagnosis, No. (%)			
Ductal carcinoma in situ, stage 0	NA	339 (22)	NA
Invasive, stage I-IV	NA	1231 (78)	

Abbreviation: NA, not applicable.

^a^ Cancer status is defined as developing ductal carcinoma in situ or invasive breast cancer after blood collection. Women with missing continuous covariate information were as follows: alcohol, 5 noncases and 1 case; physical activity, 11 noncases and 11 cases; hormone therapy, 5 noncases and 4 cases; oral contraception use, 2 noncases and 1 case; parity, 2 noncases; and menarche age, 1 noncase and 2 cases.

^b^ *P* values were calculated using 2-sample *t* tests for continuous characteristics and χ^2^ tests for categorical characteristics.

^c^ Among 2257 parous women, 2 reported at least 1 live birth but were missing age at first birth.

^d^ Among 1922 postmenopausal women, 25 reported postmenopausal status but were missing age at menopause.

^e^ Body mass index is calculated as the weight in kilograms divided by height in meters squared.

### Leukocyte Correlations and Distributions

**Figure 1.**
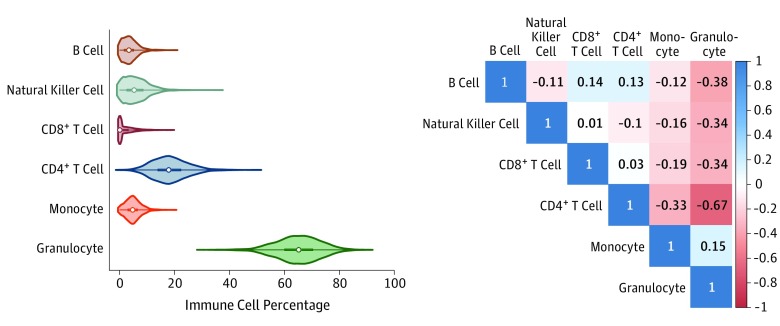
Descriptions of the Leukocyte Proportions Leukocyte subtype distributions from the random subcohort (1295 participants) are depicted as violin plots (left panel), which represent the distribution of the values (shaded portion), median (open circle), interquartile range (thick line), and 1.5 times the interquartile range (thin line). Granulocytes were the most abundant leukocyte subtype, followed by CD4^+^ T cells. A Spearman correlation matrix (right panel) shows the bivariate correlations across the 6 leukocyte subtypes (B cells, natural killer cells, CD8^+^ T cells, CD4^+^ T cells, monocytes, and granulocytes). Granulocytes were inversely correlated with the lymphocyte subtypes (B cells, natural killer cells, and CD8^+^ and CD4^+^ T cells) and were positively correlated with monocytes.

As expected, among women randomly selected from the full cohort, granulocytes composed the largest percentage of circulating leukocytes (mean [SD], 65.0% [8.1%]) and CD8^+^ T cells were the smallest percentage (mean [SD], 2.0% [3.0%]) (Figure 1). Myeloid lineage leukocytes (monocytes and granulocytes) were positively correlated with each other (*r_s_* = 0.15; 95% CI, 0.10 to 0.20; *P* < .001) and were inversely correlated with all lymphoid lineage leukocytes (Figure 1). The most significant inverse correlation was observed for granulocytes and CD4^+^ T cells (*r_s_* = –0.67; 95% CI, –0.70 to –0.63; *P* < .001).

Age had a positive correlation with natural killer cells (*r_s_* = 0.25; 95% CI, 0.20 to 0.30; *P* < .001) and inverse correlations with CD8^+^ T cells (*r_s_* = –0.18; 95% CI, –0.23 to –0.13; *P* < .001) and granulocytes (*r_s_* = –0.11; 95% CI, –0.17 to –0.06; *P* < .001) (eFigure 2 in the Supplement). Age was not significantly correlated with B cells (*r_s_* = 0.02; 95% CI, –0.03 to 0.08; *P* = .40), CD4^+^ T cells (*r_s_* = 0.02; 95% CI, –0.04 to 0.07; *P* = .52), or monocytes (*r_s_* = 0.02; 95% CI, –0.04 to 0.07; *P* = .53).

### Leukocyte Subtype Stability Over Time

In an independent sample of 8 cancer-free women, we used serially collected blood samples obtained at 3 time points, approximately 5 months apart, to examine the temporal stability of the methylation-derived leukocyte subtypes. Plots of the estimated leukocyte percentages at the 3 time points are shown in eFigure 3 in the Supplement. The leukocyte subtypes were generally stable over short periods (ICCs >0.85); however, lower ICCs were observed for monocytes and natural killer cells, suggesting some variability over the time points (monocyte ICC, 0.54; natural killer cell ICC, 0.65).

### Leukocyte Subtypes and Breast Cancer Risk

**Figure 2.**
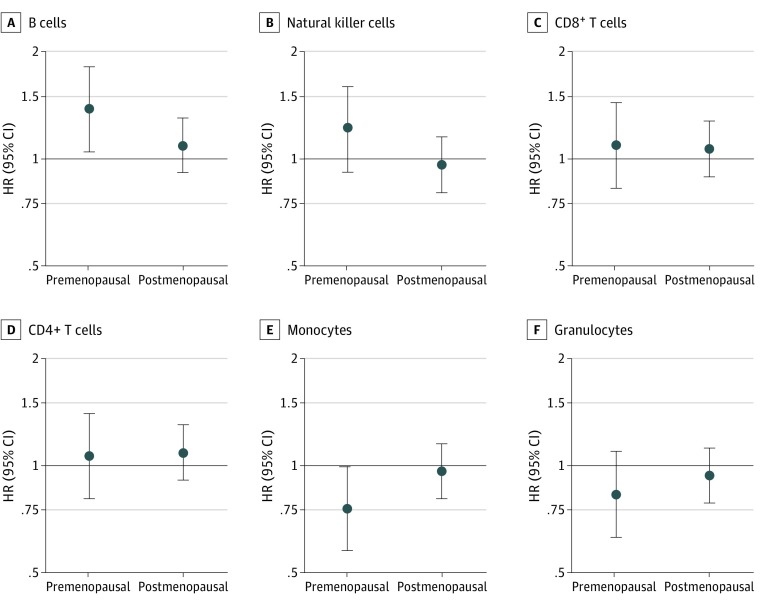
Leukocyte Proportions and Breast Cancer Risk by Menopause Status at Blood Collection Breast cancer hazard ratios (HRs) for the 6 leukocyte subtypes are shown stratified by menopause status at blood collection. Cox models were unadjusted for covariates, except for age, which was treated as the timescale. Hazard ratios (dots) and 95% CIs (error bars) are shown for women with values above vs below the median proportions. Among premenopausal women, higher monocyte proportions were associated with decreased breast cancer risk, whereas higher B-cell proportions were associated with increased breast cancer risk.

Overall, we found that women with B cell proportions greater than the subcohort median experienced higher breast cancer risk (HR, 1.17; 95% CI, 1.01-1.36; *P* = .04) (Table 2). Women with proportions of natural killer cells (HR, 1.03; 95% CI, 0.89-1.20; *P* = .70), CD8^+^ T cells (HR, 1.07; 95% CI, 0.92-1.24; *P* = .38), and CD4^+^ T cells (HR, 1.09; 95% CI, 0.94-1.27; *P* = .26) greater than the subcohort median had higher breast cancer risks, but the associations were not statistically significant. A higher mdNLR was not associated with breast cancer risk (HR, 0.91; 95% CI, 0.78-1.06; *P* = .23), nor were increases in monocytes (HR, 0.90; 95% CI, 0.77-1.04; *P* = .16) or granulocytes (HR, 0.91; 95% CI, 0.78-1.05; *P* = .20). Model adjustment for baseline covariates did not meaningfully alter the HR estimates; therefore, we focus on models without covariates (model 1) (Table 2). After stratification by menopausal status at blood collection, leukocyte subtype associations with breast cancer were primarily observed among premenopausal women, with little evidence of association among postmenopausal women (Figure 2). Specifically, we found that higher proportions of B cells were associated with increased risk of breast cancer among premenopausal women (premenopausal HR, 1.38; 95% CI, 1.05-1.82; *P* = .02; postmenopausal HR, 1.09; 95% CI, 0.91-1.30; *P* = .36; *P* for heterogeneity = 0.15), whereas higher proportions of monocytes were associated with decreased breast cancer risk among premenopausal women (premenopausal HR, 0.75; 95% CI, 0.57-0.99; *P* = .05; postmenopausal HR, 0.96; 95% CI, 0.81-1.15; *P* = .69; *P* for heterogeneity = .13) (eTable 1 in the Supplement). When we examined leukocyte subtype associations with risk of invasive breast cancer or DCIS separately, the associations were not significantly different (eTable 2 in the Supplement).

**Table 2.** Cox Proportional HRs for Leukocyte Subtypes (Comparing Above vs Below the Median Proportion) and Breast Cancer, With Age as the Timescale^a^

Variable	Model 1^b^		Model 2^c^		Model 3^d^	
	HR (95% CI)	*P* Value	HR (95% CI)	*P* Value	HR (95% CI)	*P* Value
Events, No./participants, No.	1569/2773		1569/2772		1550/2727	
Lymphocytes						
B cells	1.17 (1.01-1.36)	.04	1.16 (1.00-1.35)	.05	1.17 (1.00-1.37)	.05
Natural killer	1.03 (0.89-1.20)	.70	1.03 (0.88-1.20)	.72	1.02 (0.87-1.19)	.84
T cells						
CD8^+^	1.07 (0.92-1.24)	.38	1.07 (0.92-1.24)	.38	1.13 (0.96-1.32)	.14
CD4^+^	1.09 (0.94-1.27)	.26	1.09 (0.93-1.26)	.29	1.11 (0.95-1.30)	.18
Myelocytes						
Monocytes	0.90 (0.77-1.04)	.16	0.90 (0.77-1.05)	.17	0.87 (0.75-1.02)	.09
Granulocytes	0.91 (0.78-1.05)	.20	0.91 (0.78-1.06)	.21	0.90 (0.77-1.05)	.19
Methylation-derived neutrophil-to-lymphocyte ratio	0.91 (0.78-1.06)	.23	0.91 (0.78-1.06)	.24	0.90 (0.77-1.05)	.18

Abbreviation: HR, hazard ratio.

^a^ One woman from the random subcohort received a diagnosis of ductal carcinoma in situ and was missing follow-up time.

^b^ Model 1 is crude, with age as the timescale.

^c^ Model 2 is adjusted for baseline menopause status, with age as the timescale.

^d^ Model 3 is adjusted for breast cancer risk factors, including age at enrollment, baseline body mass index (calculated as the weight in kilograms divided by height in meters squared), menopause status, an interaction term for body mass index and menopause, physical activity, alcohol intake, parity, age at first birth (among parous women), age at menarche, breastfeeding duration, and hormone therapy and oral contraception duration, with age as the timescale.

### Time Dependency of Leukocyte Subtypes and Breast Cancer Risk

**Figure 3.**
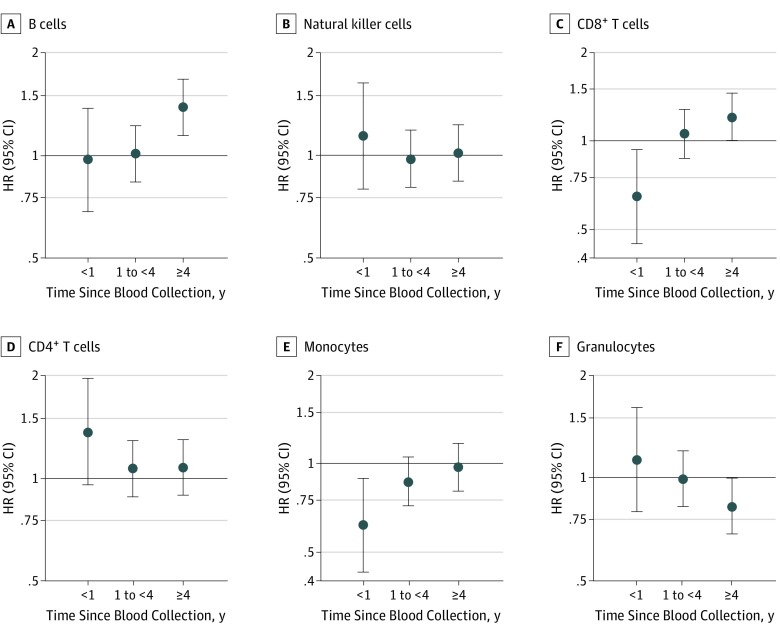
Leukocyte Proportions and Breast Cancer Risk by Time Since Blood Collection Breast cancer hazard ratios (HRs) for the 6 leukocyte subtypes are shown by time since blood collection. Cox models were unadjusted for covariates, except for age, which was treated as the timescale. Hazard ratios (dots) and 95% CIs (error bars) are shown for women with values above vs below the median proportions. Higher B-cell proportions were associated with increased breast cancer risk 4 or more years after blood collection, whereas higher monocyte proportions were associated with reduced breast cancer risk within the year following blood collection.

To examine whether the leukocyte subtype associations with breast cancer risk were time dependent, we stratified by time since blood collection. Overall, 151 women received a diagnosis of breast cancer within the first year of follow-up, 699 received a diagnosis at 1 to less than 4 years, and 719 received a diagnosis 4 years or more after blood collection. Although monocyte proportions were not associated with breast cancer development at the longer time frames, women with proportions higher than the median experienced lower risk of breast cancer in the first year of follow-up (HR, 0.62; 95% CI, 0.43-0.89; *P* = .01) (Figure 3). Women with higher B-cell proportions at blood collection had no increased risk within 1 year of blood collection or from 1 to less than 4 years after, but they had a higher risk of breast cancer 4 years or more after blood collection (HR, 1.38; 95% CI, 1.15-1.67; *P* = .001). After stratification by tumor invasiveness, the year 1 association with monocyte proportions appeared to be more significant among women with invasive tumors (invasive HR, 0.55; 95% CI, 0.36-0.84; *P* = .01; DCIS HR, 0.89; 95% CI, 0.43-1.83; *P* = .75) (eTable 3 in the Supplement). The mdNLR was inversely associated with DCIS risk, but only 4 or more years after blood collection (HR, 0.66; 95% CI, 0.47-0.94; *P* = .02) (eTable 3 in the Supplement).

Although CD8^+^ and CD4^+^ T cells had no associations overall or after stratification by menopause status, the proportions appeared to be associated with breast cancer within 1 year of blood collection (Figure 3). Such associations could be residual associations of the previously established correlation between monocytes and these 2 classes of T cells, so we explored associations using a mutually adjusted model of the leukocyte subtypes. Because the leukocyte proportions represent compositional data, we excluded natural killer cell proportions to avoid colinearity among the factors. Although the association with CD4^+^ T cells was attenuated, we found that the near-term breast cancer associations for CD8^+^ T cell and monocyte subtypes remained unchanged (eTable 4 in the Supplement). Women with elevated granulocyte or CD8^+^ T-cell proportions appeared to experience increased risk of breast cancer 4 or more years after blood collection, but these associations did not persist in the mutually adjusted model (eTable 4 in the Supplement).

## Discussion

On the basis of a case-cohort analysis of the prospective Sister Study cohort, the methylation-derived subtypes of peripheral blood leukocytes were associated with breast cancer risk. Specifically, in the years after blood collection, we found that circulating proportions of monocytes were inversely associated with breast cancer risk, whereas circulating proportions of B cells were positively associated with risk. These associations were primarily observed among premenopausal women and appeared to be time dependent: women with higher proportions of monocytes experienced lower risk of breast cancer during the first year of follow-up, whereas women with higher proportions of B cells experienced higher risk of breast cancer in the period starting 4 years after blood collection. Although the mdNLR is reported to be a marker of breast cancer prognosis,^32^ we did not find substantial evidence that it is associated with breast cancer risk. Together, our findings suggest that women’s circulating immune cell profiles may change in the years leading up to a breast cancer diagnosis.

The immune system can play paradoxical roles both protecting against and facilitating cancer development.^56,57,58^ Different subtypes of immune cells are known to play different roles at a local level during tumor development,^59,60,61^ supporting the broader hypothesis of alterations in circulating leukocyte profiles before a clinical diagnosis of a cancer. In the Women’s Health Initiative,^11^ elevated leukocyte counts were found to be associated with risk of several cancers, including breast cancer, and these associations remained largely unchanged even after exclusion of cancers that occur within 2 years of blood collection. Although methylation-based measures cannot estimate absolute leukocyte counts, our data suggest that the underlying proportions of leukocyte subtypes shift in the years preceding diagnosis.

We found that proportions of circulating monocytes were inversely associated with breast cancer risk shortly after blood collection, suggesting that lower proportions of monocytes may be a marker of increased near-term risk. This interpretation is consistent with earlier studies suggesting that circulating monocytes may be recruited to breast tumors via chemotactic signals, where they differentiate into tumor-associated macrophages.^62,63,64,65^ Breast cancer tumor–associated macrophages can further amplify recruitment signals via expression of chemokine ligand 8, creating a positive regulatory loop, potentially reducing the circulating monocyte reservoir.^65^

Women with higher proportions of B cells had no change in near-term breast cancer risk but had increased risk of breast cancer at longer intervals, 4 or more years after blood collection. B cells are a diverse set of cells best known for their role in antibody production and humoral immunity.^66^ Subsets of B cells may suppress antitumor immune responses, whereas others may provide protection.^67,68,69,70^ Although the elevation in B-cell proportion that we observed years before clinical diagnosis may reflect an early biological event in breast cancer development, we cannot rule out the possibility that this finding arose by chance. If unique methylation marks can be found, more detailed subtyping of B cells could be useful.

We also found that the leukocyte subtype associations with breast cancer were generally more significant among premenopausal women. Although the earlier study of leukocyte counts in the Women’s Health Initiative by Margolis et al^11^ was restricted to women who were postmenopausal at enrollment, Park et al^12^ recently reported that breast cancer associations with leukocyte counts were more significant among premenopausal women. Age-specific associations also have been observed between circulating inflammatory markers and breast cancer risk, perhaps reflecting immunosenescent changes with age.^71,72^ The robustness of our findings is supported by the temporal stability of the leukocyte proportions and that the leukocyte associations with age in our data are largely consistent with what has been previously reported.^73^

### Limitations

This study has limitations. Although we found time-dependent shifts in leukocyte profiles before a breast cancer diagnosis, our study was observational and we could only assess the relative proportions of leukocytes at the time when follow-up began. Thus, we could only describe associations between leukocyte subtypes and later breast cancer risk and could not assess effects of leukocyte changes or identify mechanisms. Although blood DNA methylation can be used to estimate the proportions of different leukocyte subtypes, we have no information on overall leukocyte counts. In our study, leukocyte subtypes were estimated using the Illumina HumanMethylation450 platform, which has been validated using external populations and is reported to have good reliability.^22^ Deconvolution methods based on the newer platform, the Illumina HumanMethylationEPIC array, may further improve these estimates,^74^ but data for the EPIC array were not available in our study. Inclusion of more-specific leukocyte subtypes, including regulatory T cells (a subset of CD4^+^ T cells) that are often found in the tumor microenvironment,^75^ may provide additional insights into the interplay of the immune system and breast cancer risk. Our study also includes multiple comparisons, and we did not explicitly adjust *P* values for the number of statistical tests. In addition, although we assessed potential confounding by established breast cancer risk factors that we measured, there is still the opportunity for residual confounding by mammographic density, medication use, or other unmeasured covariates.

## Conclusions

In summary, we found that the relative proportions of circulating leukocyte subtypes appeared to be associated with the risk of breast cancer months to years after blood collection. Although routine clinical white blood cell counts do not differentiate among lymphocyte subtypes, monocyte differential counts could be obtained from medical records and may be associated with near-term breast cancer risk. Moreover, in epidemiologic settings, methylation-based deconvolution provides a useful tool for high-throughput cellular phenotyping and may offer the opportunity to test immunologic hypotheses in large populations.

## Notes

Supplement. **eFigure 1.** Sampling Procedure for the Case-Cohort Subsample **eFigure 2.** Leukocyte Proportions Correlations With Age **eFigure 3.** Leukocyte Proportion Estimates Over Time **eTable 1.** Cox Proportional Hazard Ratios for Leukocyte Subtypes (Comparing Above vs Below the Median Proportion) and Breast Cancer, by Menopause Status at Blood Draw **eTable 2.** Cox Proportional Hazard Ratios for Leukocyte Subtypes (Comparing Above vs Below the Median Proportion) and Invasive Breast Cancer and DCIS Risk, Overall and Stratified by Menopausal Status at Blood Draw **eTable 3.** Cox Proportional Hazard Ratios for Leukocyte Subtypes (Comparing High vs Low Proportions) and Invasive Breast Cancer and DCIS Risk, Overall and Stratified by Time Since Blood Draw **eTable 4.** Mutually Adjusted Cox Proportional Hazard Ratios for Leukocyte Subtypes (Comparing Above vs Below the Median Proportion) and Breast Cancer Risk, by Time Since Blood Draw
