# Supplementary material for: Prediagnostic Immune Cell Profiles and Breast Cancer
Source: JAMA Netw Open. 2020 Jan 17;3(1):e1919536. doi: 10.1001/jamanetworkopen.2019.19536 (PMC6991268; doi:10.1001/jamanetworkopen.2019.19536)
Supplement: Supplement. — eFigure 1. Sampling Procedure for the Case-Cohort Subsample eFigure 2. Leukocyte Proportions Correlations With Age eFigure 3. Leukocyte Proportion Estimates Over Time eTable 1. Cox Proportional Hazard Ratios for Leukocyte Subtypes (Comparing Above vs Below the Median Proportion) and Breast Cancer, by Menopause Status at Blood Draw eTable 2. Cox Proportional Hazard Ratios for Leukocyte Subtypes (Comparing Above vs Below the Median Proportion) and Invasive Breast Cancer and DCIS Risk, Overall and Stratified by Menopausal Status at Blood Draw eTable 3. Cox Proportional Hazard Ratios for Leukocyte Subtypes (Comparing High vs Low Proportions) and Invasive Breast Cancer and DCIS Risk, Overall and Stratified by Time Since Blood Draw eTable 4. Mutually Adjusted Cox Proportional Hazard Ratios for Leukocyte Subtypes (Comparing Above vs Below the Median Proportion) and Breast Cancer Risk, by Time Since Blood Draw [file jamanetwopen-3-e1919536-s001.pdf]

Supplementary Online Content

Kresovich JK, O'Brien KM, Xu Z, Weinberg CR, Sandler DP, Taylor JA. Prediagnostic immune cell profiles and breast cancer. *JAMA Netw Open*. 2020;3(1):e1919536. doi:10.1001/jamanetworkopen.2019.19536

eFigure 1. Sampling Procedure for the Case-Cohort Subsample

eFigure 2. Leukocyte Proportions Correlations With Age

eFigure 3. Leukocyte Proportion Estimates Over Time

eTable 1. Cox Proportional Hazard Ratios for Leukocyte Subtypes (Comparing Above vs Below the Median Proportion) and Breast Cancer, by Menopause Status at Blood Draw

eTable 2. Cox Proportional Hazard Ratios for Leukocyte Subtypes (Comparing Above vs Below the Median Proportion) and Invasive Breast Cancer and DCIS Risk, Overall and Stratified by Menopausal Status at Blood Draw

eTable 3. Cox Proportional Hazard Ratios for Leukocyte Subtypes (Comparing High vs Low Proportions) and Invasive Breast Cancer and DCIS Risk, Overall and Stratified by Time Since Blood Draw

eTable 4. Mutually Adjusted Cox Proportional Hazard Ratios for Leukocyte Subtypes (Comparing Above vs Below the Median Proportion) and Breast Cancer Risk, by Time Since Blood Draw

This supplementary material has been provided by the authors to give readers additional information about their work.

© 2020 Kresovich JK et al. *JAMA Network Open.*

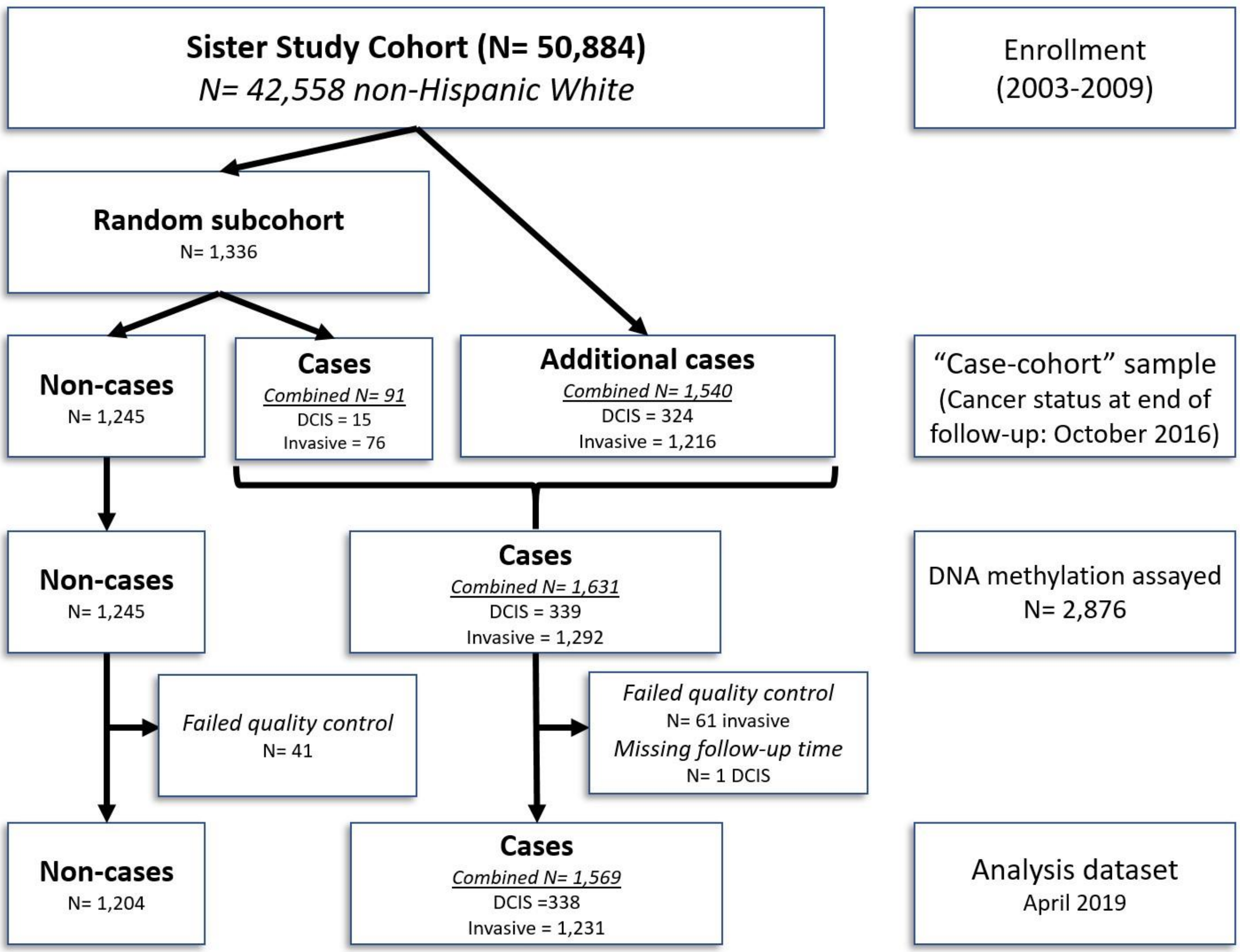

eFigure 1. Sampling Procedure for the Case-Cohort Subsample.

Of 42,558 non-Hispanic White women enrolled in the Sister Study, 1,336 were randomly sampled from the full cohort. Of these women, 91 developed breast cancer (15 ductal carcinoma *in situ* [DCIS] and 76 invasive) by October 2016. The random subsample was supplemented with an additional 1,540 cases (324 DCIS and 1,216 invasive) that developed cancer by October 2016. In total, 2,876 women were selected for blood genome-wide DNA methylation assessment. Of the 2,876 assays, 102 failed quality control: 91 samples that had average bisulfite intensity < 4000 or had more than 5% of probes with low-quality methylation values (detection p >0.000001, number of beads <3, or values outside of 3IQR); 4 samples that were outliers for their methylation beta value distributions; 1 sample with missing phenotype data; and 6 women whose date of diagnosis preceded blood draw. After quality control, 1,204 non-cases and 1,569 cases remained for analysis (conducted in April 2019).

© 2020 Kresovich JK et al. *JAMA Network Open.*

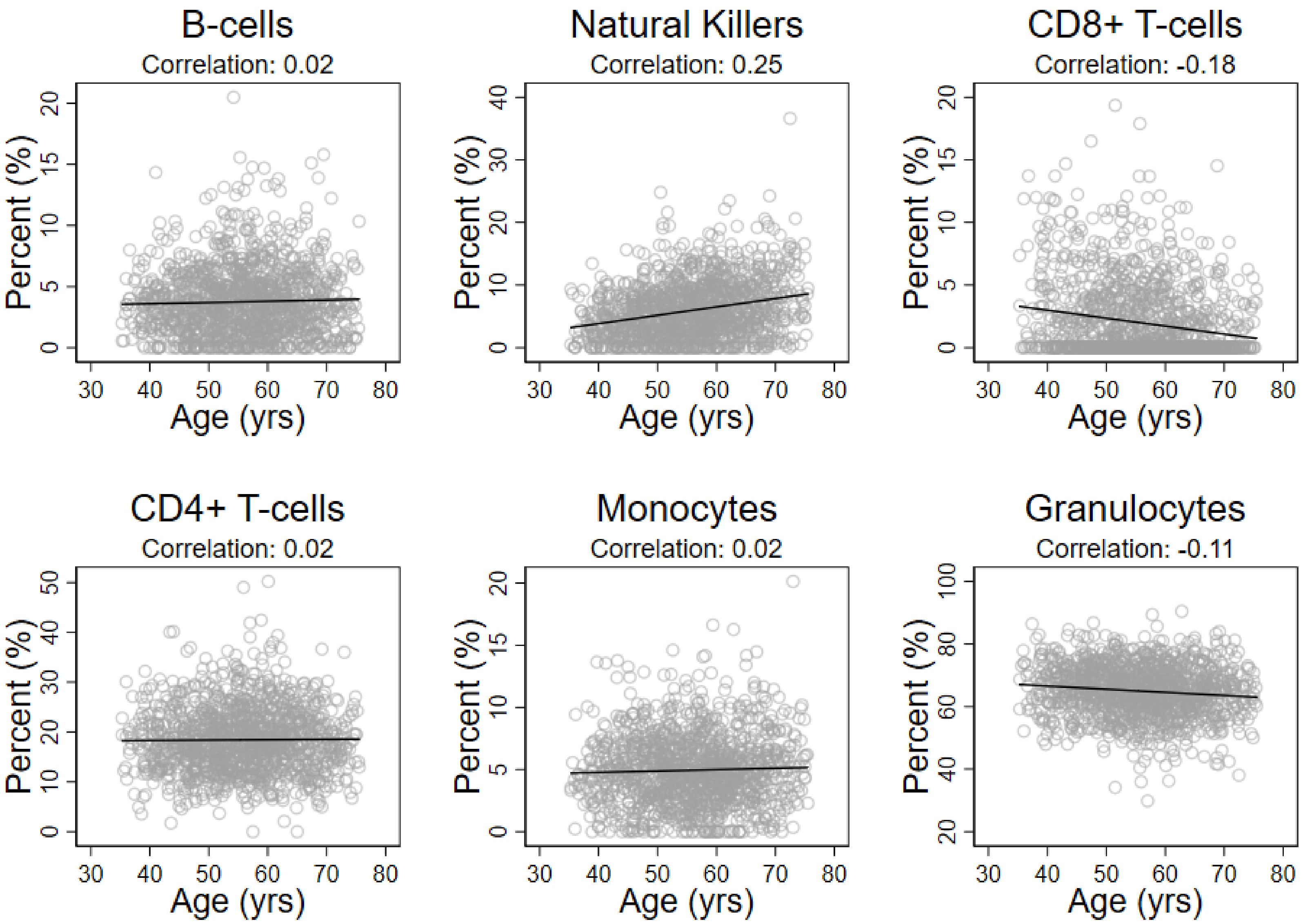

eFigure 2. Leukocyte Proportions Correlations With Age

Scatter plots and Spearman correlations for the six leukocyte subtypes and age at blood draw among women in the random subcohort (n= 1,295).

© 2020 Kresovich JK et al. *JAMA Network Open.*

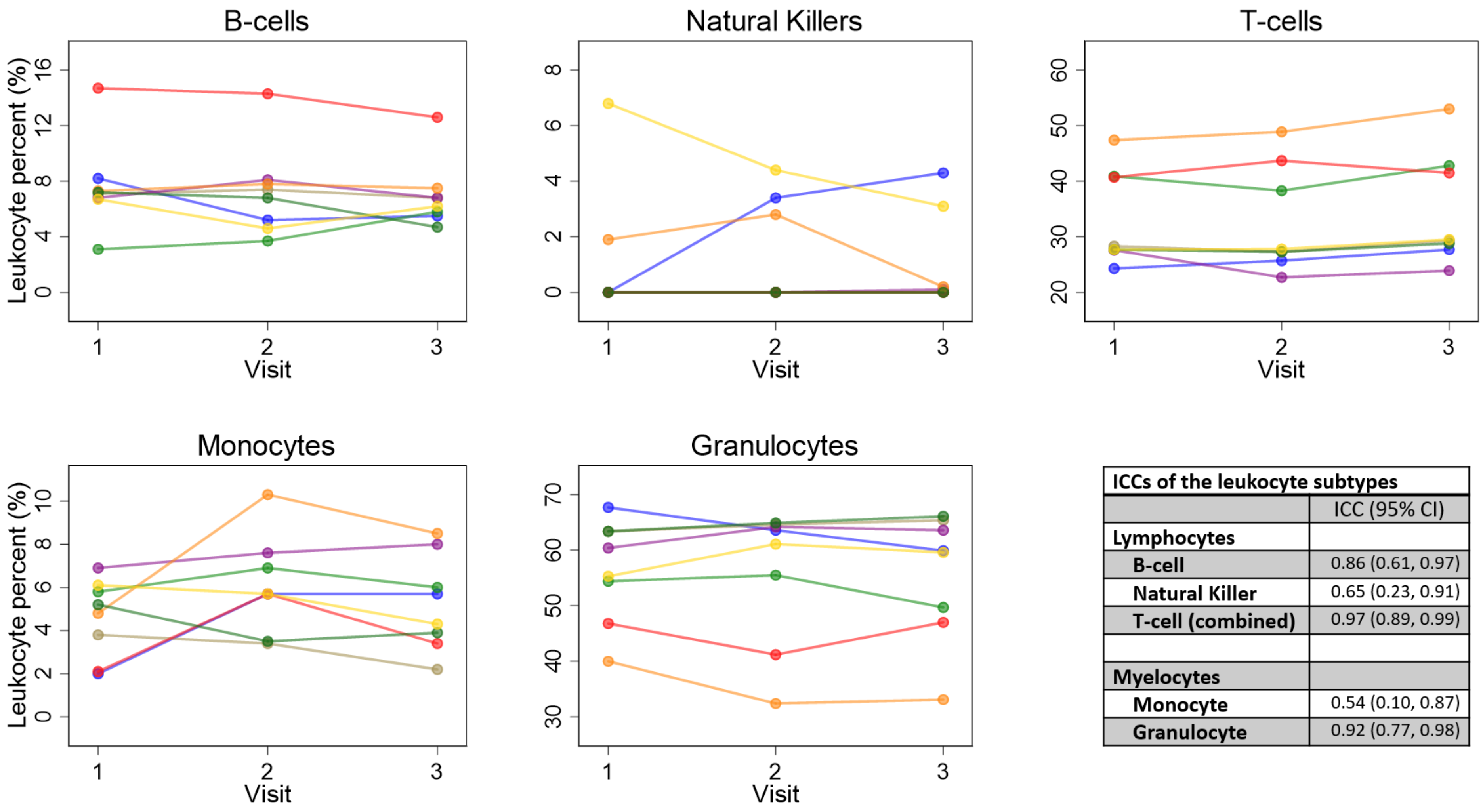

ICCs of the leukocyte subtypes	
	ICC (95% CI)
**Lymphocytes**	
**B-cell**	0.86 (0.61, 0.97)
**Natural Killer**	0.65 (0.23, 0.91)
**T-cell (combined)**	0.97 (0.89, 0.99)
**Myelocytes**	
**Monocyte**	0.54 (0.10, 0.87)
**Granulocyte**	0.92 (0.77, 0.98)

eFigure 3. Leukocyte Proportion Estimates Over Time

Leukocyte subtypes estimated among an independent sample of cancer-free women (n= 8) with three blood draws over a one-year period, approximately five months apart. Each line color represents an individual (consistent across cell types). Genome-wide DNA methylation was assessed using an older methylation array (Infinium HumanMethylaiton27 BeadChip) and CD4+ and CD8+ T-cells were combined.

© 2020 Kresovich JK et al. *JAMA Network Open.*

eTable 1. Cox Proportional Hazard Ratios for Leukocyte Subtypes (Comparing Above vs Below the Median Proportion) and Breast Cancer, by Menopause Status at Blood Draw

	Premenopausal			Postmenopausal		
	HR (95% CI)	P-value		HR (95% CI)	P-value	*P*-het
Events/subjects	418/826			1,151/1,946		
Lymphocytes						
B-cells	1.38 (1.05, 1.82)	0.02		1.09 (0.91, 1.30)	0.36	0.15
Natural Killer	1.21 (0.92, 1.60)	0.18		0.97 (0.81, 1.16)	0.70	0.24
CD8+ T-cells	1.09 (0.83, 1.44)	0.53		1.07 (0.89, 1.28)	0.47	0.87
CD4+ T-cells	1.06 (0.81, 1.40)	0.66		1.09 (0.91, 1.30)	0.35	0.90
Myelocytes						
Monocytes	0.75 (0.57, 0.99)	0.05		0.96 (0.81, 1.15)	0.69	0.13
Granulocytes	0.83 (0.63, 1.10)	0.19		0.94 (0.78, 1.12)	0.49	0.52
Ratio measures						
mdNLR	0.80 (0.60, 1.06)	0.12		0.96 (0.80, 1.15)	0.64	0.32

*Age treated as the time-scale in the Cox regression models
P-heterogeneity tested using an interaction term between menopause status at blood draw and the leukocyte subtype.
Abbreviations: hazard ratio, HR; confidence interval, CI; methylation-derived neutrophil to lymphocyte ratio, mdNLR

© 2020 Kresovich JK et al. *JAMA Network Open.*

eTable 2. Cox Proportional Hazard Ratios for Leukocyte Subtypes (Comparing Above vs Below the Median Proportion) and Invasive Breast Cancer and DCIS Risk, Overall and Stratified by Menopausal Status at Blood Draw								
	Full sample			Premenopausal			Postmenopausal	
	HR (95% CI)	P-value		HR (95% CI)	P-value		HR (95% CI)	P-value
**Invasive breast cancer**								
Events/subjects	1,231/2,449			326/737			905/1,711	
Lymphocytes								
B-cells	1.14 (0.97, 1.34)	0.11		1.36 (1.01, 1.83)	0.04		1.06 (0.88, 1.28)	0.56
Natural Killer	1.04 (0.88, 1.22)	0.67		1.15 (0.85, 1.55)	0.36		0.99 (0.82, 1.20)	0.91
CD8+ T-cells	1.06 (0.90, 1.25)	0.47		1.08 (0.80, 1.45)	0.62		1.06 (0.88, 1.29)	0.53
CD4+ T-cells	1.12 (0.95, 1.31)	0.17		1.13 (0.85, 1.52)	0.40		1.10 (0.91, 1.33)	0.31
Myelocytes								
Monocytes	0.88 (0.75, 1.04)	0.13		0.69 (0.51, 0.92)	0.01		0.97 (0.81, 1.18)	0.78
Granulocytes	0.94 (0.80, 1.10)	0.44		0.83 (0.62, 1.12)	0.23		0.98 (0.81, 1.19)	0.87
Ratio measures								
mdNLR	0.93 (0.79, 1.09)	0.34		0.80 (0.59, 1.08)	0.14		0.98 (0.81, 1.18)	0.83
**Ductal carcinoma *in situ***								
Events/subjects	337/1,617			92/516			245/1,100	
Lymphocytes								
B-cells	1.29 (1.01, 1.64)	0.04		1.47 (0.93, 2.34)	0.10		1.22 (0.91, 1.63)	0.18
Natural Killer	1.01 (0.79, 1.29)	0.96		1.46 (0.92, 2.31)	0.11		0.87 (0.65, 1.17)	0.36
CD8+ T-cells	1.11 (0.87, 1.41)	0.40		1.15 (0.72, 1.81)	0.56		1.10 (0.83, 1.46)	0.51
CD4+ T-cells	0.99 (0.77, 1.26)	0.91		0.84 (0.53, 1.34)	0.47		1.03 (0.77, 1.37)	0.84
Myelocytes								
Monocytes	0.95 (0.75, 1.22)	0.71		1.04 (0.66, 1.65)	0.86		0.93 (0.70, 1.25)	0.64
Granulocytes	0.80 (0.63, 1.02)	0.08		0.83 (0.52, 1.31)	0.41		0.79 (0.59, 1.06)	0.12
Ratio measures								
mdNLR	0.87 (0.68, 1.10)	0.24		0.81 (0.51, 1.28)	0.37		0.89 (0.67, 1.18)	0.42
Abbreviations: hazard ratio, HR; confidence interval, CI; methylation-derived neutrophil to lymphocyte ratio, mdNLR								

© 2020 Kresovich JK et al. *JAMA Network Open.*

eTable 3. Cox Proportional Hazard Ratios for Leukocyte Subtypes (Comparing High vs Low Proportions) and Invasive Breast Cancer and DCIS Risk, Overall and Stratified by Time Since Blood Draw

	< 1 year			1-4 years			4+ years	
	HR (95% CI)	P-value		HR (95% CI)	P-value		HR (95% CI)	P-value
**Invasive breast cancer**								
Events/subjects	116/1,404			543/1,797			572/1,744	
Lymphocytes								
B-cells	1.00 (0.67, 1.49)	0.99		1.02 (0.83, 1.25)	0.88		1.29 (1.05, 1.58)	0.01
Natural Killer	1.14 (0.76, 1.70)	0.53		1.00 (0.81, 1.23)	0.99		1.01 (0.82, 1.24)	0.93
CD8+ T-cells	0.62 (0.41, 0.95)	0.03		1.07 (0.87, 1.31)	0.55		1.17 (0.95, 1.43)	0.13
CD4+ T-cells	1.44 (0.95, 2.17)	0.08		1.13 (0.91, 1.39)	0.26		1.06 (0.87, 1.30)	0.56
Myelocytes								
Monocytes	0.55 (0.36, 0.84)	0.01		0.89 (0.72, 1.09)	0.25		0.94 (0.77, 1.15)	0.55
Granulocytes	1.18 (0.79, 1.77)	0.42		0.97 (0.79, 1.19)	0.77		0.90 (0.74, 1.10)	0.32
Ratio measures								
mdNLR	1.05 (0.70, 1.57)	0.82		0.96 (0.78, 1.18)	0.70		0.91 (0.74, 1.11)	0.35
**Ductal carcinoma *in situ***								
Events/subjects	35/1,325			155/1,431			147/1,355	
Lymphocytes								
B-cells	0.89 (0.45, 1.75)	0.73		1.02 (0.73, 1.42)	0.93		1.83 (1.28, 2.62)	< 0.01
Natural Killer	1.14 (0.56, 2.33)	0.71		0.89 (0.63, 1.26)	0.52		1.04 (0.73, 1.48)	0.83
CD8+ T-cells	0.72 (0.35, 1.47)	0.36		1.04 (0.74, 1.46)	0.81		1.35 (0.96, 1.90)	0.09
CD4+ T-cells	1.16 (0.56, 2.40)	0.68		0.86 (0.61, 1.21)	0.39		1.11 (0.79, 1.57)	0.54
Myelocytes								
Monocytes	0.89 (0.43, 1.83)	0.75		0.83 (0.59, 1.16)	0.28		1.11 (0.78, 1.58)	0.55
Granulocytes	0.98 (0.49, 1.96)	0.96		1.10 (0.78, 1.54)	0.60		0.56 (0.39, 0.81)	< 0.01
Ratio measures								
mdNLR	1.18 (0.57, 2.42)	0.65		1.08 (0.76, 1.52)	0.68		0.66 (0.47, 0.94)	0.02

© 2020 Kresovich JK et al. *JAMA Network Open.*

eTable 4. Mutually Adjusted Cox Proportional Hazard Ratios for Leukocyte Subtypes (Comparing Above vs Below the Median Proportion) and Breast Cancer Risk, by Time Since Blood Draw

	< 1 year			1-4 years			4+ years	
	HR (95% CI)	P-value		HR (95% CI)	P-value		HR (95% CI)	P-value
Events/subjects	151/1,435			699/1,949			719/1,885	
Lymphocytes								
B-cells	0.99 (0.68, 1.43)	0.94		1.00 (0.82, 1.21)	0.99		1.34 (1.10, 1.62)	< 0.01
Natural Killer	*Excluded*			*Excluded*			*Excluded*	
CD8+ T-cells	0.63 (0.42, 0.94)	0.02		1.05 (0.86, 1.29)	0.64		1.13 (0.92, 1.39)	0.23
CD4+ T-cells	1.38 (0.91, 2.10)	0.13		1.06 (0.85, 1.33)	0.61		1.00 (0.80, 1.25)	0.99
Myelocytes								
Monocytes	0.60 (0.41, 0.88)	0.01		0.88 (0.73, 1.07)	0.21		1.04 (0.86, 1.26)	0.70
Granulocytes	1.20 (0.79, 1.84)	0.39		1.05 (0.83, 1.33)	0.68		0.91 (0.72, 1.15)	0.44

As the leukocyte proportions represent compositional data, natural killer proportions were excluded to avoid model overfitting.

© 2020 Kresovich JK et al. *JAMA Network Open.*
